# Toxicity of Pb contaminated soils to the oribatid mite *Platynothrus peltifer*

**DOI:** 10.1007/s10646-015-1439-3

**Published:** 2015-03-11

**Authors:** Wei Luo, Rudo A. Verweij, Cornelis A. M. van Gestel

**Affiliations:** 1 State Key Lab of Urban and Regional Ecology, Research Center for Eco-Environmental Sciences, Chinese Academy of Sciences, Beijing, 100085 China; 2 Department of Ecological Science, Faculty of Earth and Life Sciences, VU University, De Boelelaan 1085, 1081 HV Amsterdam, The Netherlands

**Keywords:** Lead, Exposure, Toxicity, Oribatid mites, Bioaccumulation

## Abstract

To understand the toxicity of Pb-polluted shooting fields, oribatid mites *Platynothrus peltifer* were exposed to shooting field soils containing 47–2398 mg Pb/kg dry weight (DW) and having ${\text{pH}}_{{{\text{CaCl}}_{ 2} }}$ 3.2–6.8 and 3.8–13 % organic matter (OM). Exposures also included artificial soils with different pH and OM contents as well as two natural soils used as controls. Exposures lasted for 2 (acute) and 12 weeks (chronic). Survival, reproduction and uptake of Pb in the mites were related to total, water-extractable and 0.01 M CaCl_2_-extractable and porewater Pb concentrations as well as soil characteristics. After both the acute and chronic exposures, adult survival was not significantly affected, while upon chronic exposure reproduction was remarkably reduced in the acidic forest soils with Pb concentrations ≥2153 mg/kg DW and ${\text{pH}}_{{{\text{CaCl}}_{ 2} }}$ ≤ 3.5. *P. peltifer* juvenile numbers were significantly negatively and internal Pb concentrations in the mites were significantly positively related with total, extractable and porewater Pb concentrations. This study shows that *P. peltifer* is not very sensitive to Pb and therefore may not be a suitable indicator of Pb-polluted soils.

## Introduction

Lead (Pb) is ranked as the number two priority hazardous substance by the US EPA (Chen et al. 2002). Annual deposition of metallic lead at shooting ranges has reached 6000 tons in the Netherlands, Finland, Denmark, Canada, England and USA (Manninen and Tanskanen 1993; Scheuhammer and Norris 1995). In recent years, shooting ranges are under increasing scrutiny as a potentially significant source of lead contamination (Chen et al. 2002; Ming et al. 2012; Murray et al. 1997; Rooney et al. 1999).

Routine practices for investigating the exposure and risk of Pb-contaminated sites involve determining total Pb concentrations in soils. However, total Pb concentrations may not predict adverse effects accurately (Ming et al. 2012; Peijnenburg et al. 1999), because exposure expressed as total Pb does not consider the effect of soil-modifying factors like pH, OM content and cation exchange capacity on Pb bioavailability (Allen 2002; Lanno 2003). Furthermore, a successful assessment of metal polluted soils also requires a battery of biological exposure tests including different soil invertebrates representative of the soil ecosystem (Hopkin 1989).

Among the soil invertebrates, oribatid mites are the world’s most numerous arthropods. They play important roles in the forming of soil structure, decomposition processes and detrital food-webs (Crossley 1977; Krantz and Walter 2009; Lebrun 1979). *Platynothrus peltifer* is a representative of the oribatid mites (Van Gestel and Doornekamp 1998). However, up to now, it has not been used so often in the assessment of metal-contaminated sites and little is known about its sensitivity to metal-polluted soils (Van Gestel and Doornekamp 1998; Van Gestel et al. 1995).

Here, the main purpose of this study is to assess sensitivity of *P. peltifer* after being acutely and chronically exposed to different field-contaminated soils from a shooting range. More specific aims were to determine survival and reproduction of *P. peltifer* following exposure to shooting range soils and relate effects to Pb uptake in the animals as well as to total and available concentrations in the soil and to soil properties. Recently, we have studied Pb bioaccumulation and toxicity to earthworms (Luo et al. 2014c), springtails (Luo et al. 2014a) and enchytraeids (Luo et al. 2014b) exposed to shooting field soils. We hypothesized that these shooting field soils would also be toxic to *P. peltifer.* We also hypothesized that toxicity would be related to the bioavailable Pb concentrations in soil, as reflected by Pb uptake in the animals. In addition, we expected toxicity to increase with increasing exposure time and to be affected by soil properties, with soil pH and organic matter (OM) content playing a major role. An additional aim is to assess suitability of this species for assessing the toxicity of field-contaminated soils, alone or as part of a battery of soil toxicity tests. It was hypothesized that this species would be a suitable indicator of Pb-polluted field soils. This paper is a part of suite of studies that aimed at linking chemical and biological measures of Pb bioavailability in shooting range soils with different landscapes, containing different concentrations of Pb to test their toxicities to different soil animals. To our knowledge, it is novel combining environmental chemistry of naturally aged shooting field soils with bioassays using *P. peltifer* for assessing Pb polluted soils.

## Materials and methods

### Soil sampling

Six natural soils were taken from three landscapes (F: forest; G: grassland; B: bullet plot) of a shooting field in the Netherlands. Assuming it had similar soil properties without being contaminated, a soccer field soil (S) near the shooting range was sampled as a reference for the survival and reproduction tests. Three artificial soils (R1, R2, R3) were prepared to resemble the shooting field soils in pH and OM content, based on OECD artificial soil (OECD 1984). R1, the standard artificial soil, was prepared with 10 % finely ground sphagnum peat (<1 mm), 20 % kaolin clay, and 70 % quartz sand (DW), adjusted with CaCO_3_ to nominal ${\text{pH}}_{{{\text{CaCl}}_{ 2} }}$ 6.0 ± 0.5. The other two artificial soils were prepared with peat contents of 5 % (R2) or 2.5 % (R3) and ${\text{pH}}_{{{\text{CaCl}}_{ 2} }}$ adjusted to nominal 3.5 (R2) or 6.5 (R3) with CaCO_3_ (Luo et al. 2014c). The standard natural LUFA 2.2 soil (LUFA-Speyer, Sp 2121, Germany) was used as an additional control of the performance of the test animals (CK).

### Soil analysis

A detailed description of soil analysis and soil characteristics was given by Luo et al. (2014c).

### Toxicity tests

Toxicity tests followed the principles of the method described by Van Gestel and Doornekamp (1998), with some modifications. *P. peltifer* were extracted with a Tullgren apparatus from leaf litter sampled in a non-polluted coniferous and oak forest in the Spanderswoud near Hilversum, the Netherlands. A plastic ring (diameter 5 cm, height 3.5 cm), closed with gauze (mesh size 1 mm) on the bottom which was sealed with a piece of plastic foil, was used as the test container. The top was a closed lid. With the aid of a microscope, ten adult *P. peltifer* were selected randomly from the extracted mites and transferred into each of ten replicate test containers filled with 15 g test soil moistened to 50 % of the maximum water holding capacity. All exposures were kept in a climate room at 20 °C, 75 % relative humidity and 16/8 h light/dark regime. The water content of the soils was checked once per week by weighing the containers and the mites were fed every 2 weeks by placing 0.1 g green algae on the soil surface. After 2 weeks, a complete series of five replicate test containers of each test soil were placed in the Tullgren for 2–3 days to extract surviving mites. This procedure was repeated after 12 weeks, to assess survival and the number of juveniles produced. The freeze-dried surviving adult mites were individually digested in a 300 μl HClO_4_/HNO_3_ mixture (1:7 v/v; Ultrex grade, Baker) and analysed for body Pb concentrations using a Perkin Elmer 5100 Atomic Absorption Spectrometer equipped with a graphite furnace assembly. Quality of the analysis was controlled by analyzing certified reference material (Dolt 4) and Pb recoveries were 100 ± 15 %.

### Data analysis

All data were tested for normality (Kolmogorov–Smirnov test) and variance homogeneity. Apart from soil physicochemical properties and toxicity data, all metal concentrations were log-transformed to achieve normal distribution. The data of soils and bioassays in different shooting fields were analyzed by one-way analysis of variance (ANOVA) and a post hoc Tukey HSD test was used to find differences among means. Pearson’s correlation coefficients (r) were used to elucidate the latent relationships between toxicity and soil physicochemical properties and (bio) available Pb concentrations. EC_10_ and EC_50_ values for effects on reproduction were estimated with a log-logistic model (Haanstra et al. 1985), applying the modification described by Van Brummelen et al. (1996). The bioaccumulation factor (BAF) was calculated as the ratio of the measured Pb concentrations in the surviving mites and the total Pb concentrations in the test soils. Statistical analysis was performed using SPSS21.0 for Windows. The significance level was set at *p* value 0.05.

## Results and discussion

### Lead extractions and soil properties

The forest soils were acidic (${\text{pH}}_{{{\text{CaCl}}_{ 2} }}$ 3.2–3.5) while the grassland soils were neutral (${\text{pH}}_{{{\text{CaCl}}_{ 2} }}$ 6.5–6.8). The highest total (2153–2398 mg/kg), water- (11.7–14.6 mg/kg) and 0.01 M CaCl_2_-extractable (279–313 mg/kg) as well as porewater Pb concentrations (12.9–13.1 mg/L) were observed in forest soils F1 and F3. For a detailed description of soil properties and metal concentrations and a discussion on the influence of soil properties on the availability of Pb in the test soils it can be referred to our earlier paper (Luo et al. 2014c).

### Toxicity tests

**Fig. 1:**
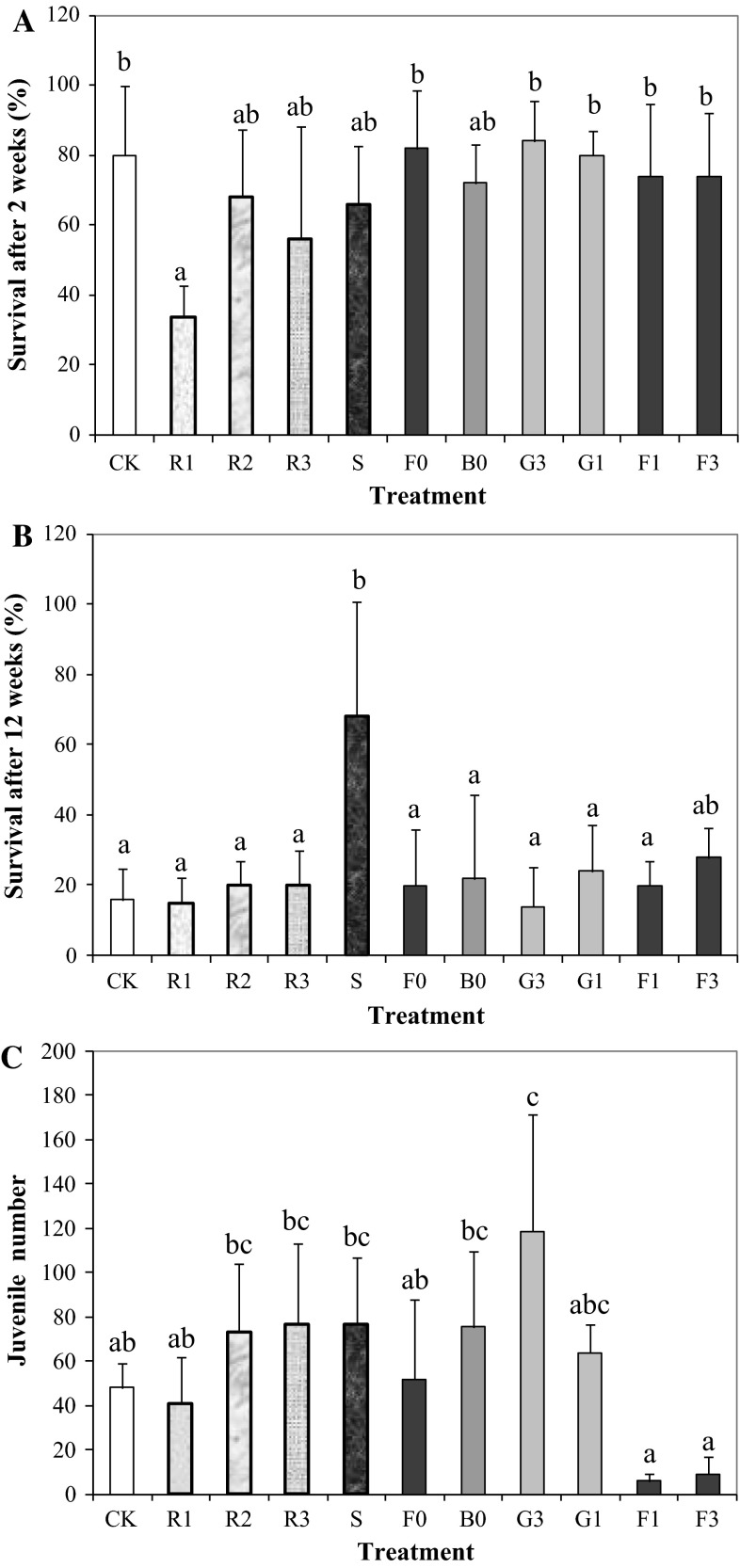
Survival of *Platynothrus peltifer* after 2 (**a**) and 12 (**b**) weeks and reproduction (**c)** after 12 weeks exposure to shooting field soils from different landscapes (*F* forest, *G* grassland, *B* bullet plot) and a reference site (*S* soccer field near shooting range) in the Netherlands and in artificial soils (R) as well as a control soil (CK: LUFA 2.2). *Columns* with the *same letter* indicate no significant differences at *p* > 0.05. Soils are arranged according to increasing total Pb concentration. See Luo et al. (2014a, b, c) for the soil codes, soil properties and metal concentrations. *Error bars* show standard deviation (*n* = 5)

After 2 weeks of exposure, the highest survival of adult *P. peltifer* (80–84 %) was observed in the grassland soils (G1, G3) and forest soil F0, while the soccer field soil had the lowest survival (66 %) among field soils (Fig. 1a). Except for R1, there was no significant difference in mite survival among field soils, artificial soils and CK (*p* > 0.05). After 12 weeks exposure, the highest survival of *P. peltifer* (68 %) was found in the soccer field soil (Fig. 1b) while in the other soils survival was significantly lower at ≤28 %. It seems that Pb concentrations as high as 2398 mg/kg DW in the soils had no impact on mite survival after 2 and 12 weeks exposure. The LC_50_ for the effect for Pb on the survival of the *Oppia nitens* after 28–35 days exposure was 6761 mg/kg DW (Owojori and Siciliano 2012), confirming the relative insensitivity of oribatide mite survival to Pb. Since the optimum pH for *P. peltifer* was 7.6, it was classified as alkalophilous and a bioindicator for soil acidification (Van Straalen and Verhoef 1997). However, in our study, the survival of *P. peltifer* in grassland soils, S and R3, which had ${\text{pH}}_{{{\text{CaCl}}_{ 2} }}$ ≥ 6.4, was not significantly greater than in the acidic soils including all forest soils, B0 and R2, which had ${\text{pH}}_{{{\text{CaCl}}_{ 2} }}$ ≤ 3.8. The absence of a significant relationship between survival and soil pH after 2 and 12 weeks exposure (Table 1) also suggests that soil pH had little impact on the mites. Therefore, we could conclude that survival of *P. peltifer* may not be a suitable endpoint for assessing Pb-contaminated shooting field soils.

**Table 1:** Simple linear correlation coefficients relating the response of *Platynothrus peltifer* to the physicochemical properties of different shooting range field soils and reference soils

Soil physicochemical properties	Simple linear correlation coefficients (*r*)			
	Survival number after 2 weeks	Survival number after 12 weeks	Juvenile number after 12 weeks	Pb in mites
WHC	−0.36**	0.50**	−0.13	−0.24
OM	−0.17	0.60**	−0.085	−0.14
pH H_2_O	−0.081	0.18	0.43**	−0.34*
pH 0.01 M CaCl_2_	−0.15	0.25	0.43**	−0.40**
DOC	−0.14	0.15	−0.27*	−0.34*
CEC	−0.15	0.49**	0.32*	−0.47**
Ca	−0.15	0.33*	0.38**	−0.48**
Fe	0.45**	0.046	−0.003	0.41**
Clay	−0.38**	−0.008	0.19	−0.57**
Silt	−0.24	−0.088	0.31*	−0.41**
Sand	0.34*	0.040	−0.25	0.53**
Pb-Water	0.039	−0.21	−0.45**	0.88**
Pb-0.01 M CaCl_2_	0.10	−0.10	−0.49**	0.82**
Pb-pore water	0.28*	−0.27*	−0.44**	0.92**
Total Pb	0.23	−0.17	−0.25	0.74**
Total Cd	0.55**	−0.19	0.38**	0.016
Total Zn	0.35**	0.087	0.29*	0.036
Total Cu	0.45**	0.029	0.21	0.26

See Luo et al. (2014a, b, c) for soil properties and metal concentrations and Fig. 1 for the mite responses and internal Pb concentrations in the mites

* Correlation is significant at the 0.05 level (2-tailed)

** Correlation is significant at the 0.01 level (2-tailed)

*Platynothrus peltifer* reproduction varied greatly, with mean total juvenile numbers ranging from 6.2 to 119 per test jar (Fig. 1c). The most contaminated forest soils F1 and F3 had the lowest juvenile numbers, indicating that both total Pb concentrations ≥2153 mg/kg DW and low ${\text{pH}}_{{{\text{CaCl}}_{ 2} }}$ ≤ 3.2 could significantly inhibit its reproduction. Since there were no significant differences in juvenile numbers between CK and artificial soils (Fig. 1c), low soil ${\text{pH}}_{{{\text{CaCl}}_{ 2} }}$ (3.8–6.4) and OM contents (2.4–8.8 %) did not significantly impact upon reproduction after 12 weeks exposure. This is confirmed by Owojori and Siciliano (2012) who found no significant difference in reproduction of the mite species *O. nitens* at soil pH levels of 3.1 and 6.1. Juvenile numbers were significantly and negatively correlated with total, extractable and pore-water Pb concentrations (*p* < 0.01) (Table 1). EC_50_s for the effect of Pb on the reproduction of *P. peltifer* were estimated to be 696, 5.5 and 48.8 mg/kg DW when related to total, water- and CaCl_2_-extractable Pb concentrations, respectively, and 6418 μg/L based on porewater Pb concentrations. Corresponding EC_10_s values were 658, 2.2 and 7.2 mg/kg DW and 3040 μg/L, respectively. No confidence intervals could be calculated for any of these EC_x_ values.

Compared to survival after 2 and 12 weeks, reproduction is a more sensitive criterion and, thus, a more suitable endpoint for assessing shooting field soils. This conclusion is in agreement with Denneman and Van Straalen (1991), Lebrun and Van Straalen (1995) and Van Straalen et al. (1989). Nevertheless, *P. peltifer* seems more tolerant to Pb than other species; all EC_50_ and EC_10_ values are higher than the ones found for the earthworm *Eisenia andrei* and the enchytraeid *Enchytraeus crypticus*, also exposed to the same soils (Luo et al. 2014b, c). Only the springtail *Folsomia candida* was less sensitive, with EC_50_ higher than the highest concentration measured in these shooting field soils (Luo et al. 2014a).

### Bioaccumulation

**Fig. 2:**
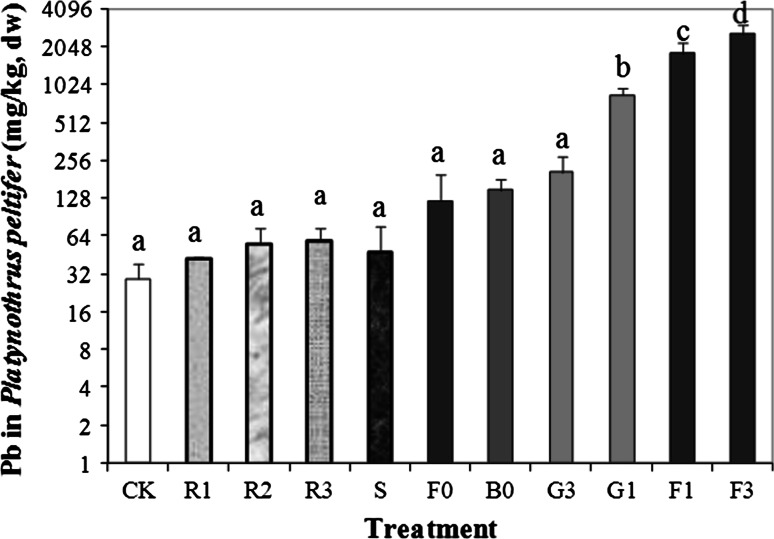
Tissue Pb concentrations in *Platynothrus peltifer* after 12 weeks exposure to shooting field soils from different landscapes (*F* forest, *G* grassland, *B* bullet plot) and a reference site (*S* soccer field near shooting range) in the Netherlands and in artificial soils (R) as well as a control (CK: LUFA 2.2). *Columns* with the *same letter* indicate no significant differences at *p* > 0.05. Soils are arranged according to increasing total Pb concentration. See Luo et al. (2014a, b, c) for the soil codes, soil properties and metal concentrations. *Error bars* show standard deviation (*n* = 5)

Lead concentrations in *P. peltifer* increased with increasing soil total Pb concentrations (Fig. 2), and also more or less linearly increased with the administered lead concentration in food (Denneman and Van Straalen 1991). The bioaccumulation factors for Pb in the mites from most soils of our study were higher than 1, indicating *P. peltifer* is an accumulator of lead. The bioaccumulation factors of Pb in *P. peltifer* in our study were obviously higher than those in *P. peltifer* collected from litter layers of forest and zinc smelter in the Netherlands (Janssen and Hogervorst 1993). Pb concentrations in the mites exposed to the three most Pb polluted soils (G1, F1, F3) were significantly higher than those kept in the other soils. This further indicates that bioaccumulation of Pb was significant at soil Pb concentrations ≥656 mg/kg DW. The high concentrations of Pb in *P. peltifer* exposed to the forest soils F1 and F3 may be explained by the high porewater Pb concentrations, which confirms exposure is mainly from the pore water (Van Gestel et al. 1995). Since the availability of Pb increases with reduced pH (Alexander 2000), the high bioavailability of Pb in the forest soils was also due to their low pH. Pb concentrations in the mites were significantly and positively correlated with total and extractable Pb concentrations in the soils and with porewater Pb concentrations, but also affected by soil texture, Fe and Ca concentrations, CEC and soil ${\text{pH}}_{{{\text{CaCl}}_{ 2} }}$ (*p* < 0.01) (Table 1).

## Conclusion

Survival of *P. peltifer* was less sensitive to Pb-contaminated shooting range soils than reproduction, making the latter a more suitable endpoint for assessing contaminated field soils. Reproduction and Pb concentrations in the mites significantly correlated with total, extractable and porewater Pb concentrations in the soils. *P. peltifer* however, seems more tolerant to Pb than other species, with effects on reproduction only occurring in acidic forest soils (${\text{pH}}_{{{\text{CaCl}}_{ 2} }}$ ≤ 3.5) with Pb concentrations ≥2153 mg/kg DW. As a consequence, this species seems less suitable for assessing the toxicity of Pb contaminated field soils.
